# Normal mammary development and function in mice with *Ift88* deleted in MMTV- and K14-Cre expressing cells

**DOI:** 10.1186/2046-2530-3-4

**Published:** 2014-03-04

**Authors:** Elizabeth H Mitchell, Rosa Serra

**Affiliations:** 1 Department of Cell, Developmental, and Integrative Biology, University of Alabama at Birmingham, 1918 University Blvd., 660 MCLM, Birmingham, AL, 35294-0005, USA

**Keywords:** Primary cilia, Intraflagellar transport, Mammosphere, Stroma, Orpk

## Abstract

**Background:**

Primary cilia (PC) are non-motile microtubule based organelles present on almost every cell type and are known to serve as critical organizing centers for several signaling pathways crucial to embryonic and postnatal development. Alterations in the Hh pathway, the most studied signaling pathway regulated by PC, affect mammary gland development as well as maintenance of the stem and progenitor cell populations.

**Results:**

We developed mouse models with deletion of PC in mammary luminal epithelial, basal epithelial, and stromal cells for evaluation of the function of PC in mammary development via MMTV-Cre, K14-Cre, and Prx1-Cre mediated deletion, respectively. The activity of Cre was confirmed using ROSA26 reporters. Mammary stem and progenitor cells were enriched through growth as mammospheres. Adenovirus-Cre mediated deletion of *Ift88* was used to determine a role for PC in this population of cells. Disruption of *Ift88* and PC were confirmed in using PCR and immunofluorescent methods. Prx1-Cre; *Ift88*^Del^ mice demonstrated defects in terminal end buds during puberty. However, these *Ift88*^Del^ glands exhibited typical terminal end bud formation as well as normal ductal histology when transplanted into wild type hosts, indicating that the phenotype observed was not intrinsic to the mammary gland. Furthermore, no discernable alterations to mammary development were observed in MMTV-Cre- or K14-Cre; *Ift88*^Del^ lines. These mice were able to feed and support several litters of pups even though wide spread depletion of PC was confirmed. Cells grown in mammosphere culture were enriched for PC containing cells suggesting PC are preferentially expressed on mammary stem and progenitor cells. Deletion of *Ift88* in mammary epithelial cells resulted in a significant reduction in the number of primary mammospheres established; however, there was no effect on outgrowth of secondary mammospheres in PC-depleted cells.

**Conclusions:**

PC regulate systemic factors that can affect mammary development in early puberty. PC on MMTV- or K14-expressing epithelial cells are not required for normal mammary development or function. PC are expressed at high levels on cells in mammosphere cultures. PC may be required for cells to establish mammospheres in culture; however, PC are not required for renewal of the cultures.

## Background

Mammary gland development is unique in that most growth occurs post-natal with terminal end bud (TEB) formation and ductal extension beginning at puberty. In adulthood, the gland undergoes cyclical restructuring during pregnancy, lactation, and involution. Mammary development is highly controlled with all cell types working together. Coordinated crosstalk between epithelial cells and stroma allows for proper terminal end bud formation, ductal extension, and side branching [1,2].

Primary cilia (PC) are organelles that were once thought insignificant but are now renowned as regulators of development and homeostasis [3-6]. These non-motile, microtubule based organelles are present one per cell on many cell types and project into the microenvironment where they serve as a signaling center for the cell, functioning as chemical and mechanical sensors. PC are formed and maintained by intraflagellar transport (IFT), a sophisticated process with large protein complexes employed in carrying cargo from the ciliary base to tip (anterograde) and tip to base (retrograde) [7]. Disruption of IFT prevents the formation, maintenance, and function of PC. Defects in genes important for the formation or function of PC have been linked to a wide array of human diseases and syndromes now termed ciliopathies [8].

The primary cilium is accepted as a critical regulator of several pathways including, Hedgehog (Hh), Wnt, PDGF, and von-Hippel Lindau tumor suppressor. The most studied of these is the Hh pathway, which is also known to be key in embryonic development and tissue homeostasis [4,7,9,10]. Hh signaling begins by the binding of one of three hedgehog secreted ligands; Sonic hedgehog (Shh), Indian hedgehog (Ihh) or Desert Hedgehog (Dhh), to its receptor patched (Ptch1) relieving its inhibition of smoothened (Smo). Smo is then able to move into the PC and mediate activation of Gli transcription factors, thus PC are required for ligand-mediated Hh signaling [7,11-14]. In the absence of Hh ligand, Gli3 is processed to a repressor form (Gli3R) that inhibits expression of transcriptional targets of Hh. PC are also required for processing Gli3 to this repressor form, therefore, PC are required for both ligand-dependent Hh signal activation as well as ligand-independent repression.

Markers of active Hh signaling are not present in normal mammary epithelium and active Hh signal in the epithelial compartment does not appear to be required for normal mammary development [15]. However, there is evidence that activation of Hh signaling in the stromal compartment is required for normal development [16]. During embryonic stages of mammary development it is likely that repression of Hh signaling is required to promote development of mammary glands rather than hair follicles, which require Hh signaling [17]. The Gli3R function is critical for embryonic mammary development and activation of Hh signaling via loss of Gli3R results in loss of mammary buds [15]. In post-natal mice, inappropriate activation of Hh signaling in mammary epithelium results in impaired mammary development and ductal dysplasia suggesting suppression of Hh signaling is critical throughout mammary development and activation could participate in formation of breast cancer [18]. A role for primary cilia in basal cell carcinoma and medulloblastoma have been demonstrated [19,20]. Furthermore, PC are differentially presented in numerous carcinomas, including; basal cell, brain, pancreas, kidney, and breast [19-23]. In the breast, PC are more frequently found on normal epithelial cells than on cancer cells. PC are also more frequent on epithelial cell lines derived from benign breast than those from breast cancer regardless of the level of proliferation in those cells suggesting PC could act as tumor suppressors [21].

In this study, we generated mice with depletion of PC via deletion of *Ift88*, an IFT protein required for formation and maintenance of PC, in specific cell types within the mammary gland. Luminal, basal, and stromal cells were targeted using MMTV-, K14-, and Prx1-Cre lines, respectively. As previously reported, we found that deletion of *Ift88* in Prx1-Cre expressing mice resulted in loss of TEB and delayed extension of ducts through the fat pad during early puberty [24]. In this study, whole gland transplant indicated that the phenotype was not inherent to the mammary gland suggesting the involvement of systemic factors. Surprisingly, alterations in mammary development or function associated with depletion of PC in MMTV-Cre or K14-Cre expressing cells were not found. Using mammosphere cultures to select for stem and progenitor cells, we found that PC are enriched on these cell populations. Depletion of primary cilia on mammary epithelial cells resulted in reduced ability of the cells to form primary mammospheres; however, formation of secondary mammospheres was not affected. We conclude that PC do not play a major role in regulating normal post-natal mammary development or function.

## Methods

### Animals

*Ift88*^orpk^, *Ift88*^LoxP/LoxP,^ K14-Cre, and MMTV-Cre mice have previously been described [25-28]. MMTV-Cre mice were obtained from the NCI mouse repository (strain 01XA9). K14-Cre, Gt(ROSA)26Sor^tm4(ACTB-tdTomato,-EGFP)Luo^/J (ROSA26^mTmG^), and Gt(ROSA)26Sor^tm1Sor^/J (Rosa26^LacZ^) mice were obtained from (Jackson Laboratories (stock 004782, 007576, and 003474), *Ift88*^orpk^ and *Ift88*^LoxP/LoxP^ mice were generously provided by Dr. Bradley Yoder University of Alabama at Birmingham. MMTV-Cre and K14-Cre mice were on a C57Bl/6 genetic background. *Ift88*^LoxP/LoxP^ mice were on a 129P2/OlaHsd background. We used two sets of *Ift88*^Orpk^ mice; either on a C57Bl/6 or a Balb/c background. Experimental crosses were set up as MMTV- or K14-Cre; *Ift88*^LoxP/Wt^ × *Ift88*^LoxP/LoxP^ to generate Cre-positive *Ift88*^LoxP/LoxP^ mice (hereafter called *Ift88*^Del^). MMTV- or K14-Cre; *Ift88*^LoxP/Wt^ and Cre-negative; *Ift88*^LoxP/LoxP^ mice were used as controls. Age matched or littermate controls were used. All mice utilized in this study were maintained following the guidelines of the Institutional Animal Care and Use Committee of the University of Alabama at Birmingham. All animal usage in this study was approved by the Institutional Animal Care and Use Committee of the University of Alabama at Birmingham.

### Beta-galactosidase staining

Mammary glands were removed and fixed in 4% paraformaldehyde (PFA) for 1 hour. Afterwards they were washed 3 times in rinse buffer (2 mM MgCl_2_, 0.01%Na Deoxycholate, 0.02%NP-40, in PBS) and stained overnight at room temperature in 1 mg/ml 4-chloro-5-bromo-3-indoyl-β-D-galactopyranoside (X-gal), 5 mM K_3_Fe(CN)_6_,5 mM K_4_FE(CN)6H_2_0, 2 mM MgCl_2_, in PBS. Mammary glands were rinsed in PBS and post fixed for one hour with 4% PFA, then dehydrated through graded ethanol and cleared in 80% glycerol before mounting. For sections, glands were equilibrated with 30% sucrose and then equilibrated with Optimal Cutting Temperature (O.C.T.) Compound (Sakura, Torrance, Ca). Glands were flash frozen in O.C.T. by liquid nitrogen in a 2-methyl butane bath. Sections were cut at 20-40 μm.

### Immunofluorescence and cilia staining

Mammary glands were removed and fixed in 4% paraformaldehyde (PFA) for 1 hour at room temperature then they were placed in 30% sucrose overnight and equilibrated with an equal amount O.C.T. After equilibration, glands were flash frozen in O.C.T by liquid nitrogen in a 2-methyl butane bath. Sections were cut at 20-40 μm and fixed in ice-cold methanol for 20 minutes. Sections from ROSA26^mTmG^ mice were stained with DAPI. Immunofluorescent staining of PC was done as described previously [29]. Slides were blocked for 1 hour with PBS with 0.1% Triton-x-100, 3% BSA, 1% Normal goat serum (Vector labs, S-1000). Primary antibodies were anti-Arl13b used at 1:2000 (a gift of Dr. Tamara Caspary, Emory University, Atlanta, GA, USA) staining was done as previously described [30] anti-actin α-smooth muscle-Cy3 (Sigma, C6198) used at 1:1000, primary antibodies incubations were overnight at 4°C. Anti-Rabbit AlexaFluor-488 (Life Technologies, A-11008) secondary was used at 1:1000. Nuclei were stained with DAPI.

### Cilia counting

20-40 μm sections were imaged using a Hamamatsu C9100-50 EM-CCD camera (Hamamatsu Photonics K.K., Hamamatsu City, Japan) on an inverted Nikon TE2000-U microscope equipped with a 60× Plan Apochromat oil-immersion TIRFM objective (numerical aperture (NA), 1.49; Nikon Instruments Inc., Melville, NY), and a Perkin Elmer Ultraview-ERS 6FE spinning disk confocal module controlled by Volocity 6.2 software (Perkin Elmer, Shelton, CT, USA). Cells positive for smooth muscle actin were considered myoepithelial cells and cells negative for smooth muscle actin but on the luminal side of the duct were considered luminal epithelial cells. Only cilia larger than 1 μm were counted. Cilia were counted on >600 luminal cells (n = 3 separate mice each for MMTV-Cre; *Ift88*^*Del*^ and controls) with at least 200 cells counted per mouse. Cilia were counted on >500 myoepithelial cells (n = 2 K14-Cre; *Ift88*^*Del*^ and n = 3 controls) with at least 150 cells counted per mouse. *T*-Test statistics were done using Microsoft Excel.

### PCR

Mammary epithelial cells were isolated as described previously [31]. Mammary glands minus lymph nodes were minced and digested with 1 mg/ml Collagenase Type I (Sigma- Aldrich, C9891, St. Louis, MO, USA) and 0.1 mg/ml Pronase (Roche Diagnostics, 1149643001, Mannheim, Germany) at 37°C in Hanks Balanced Salt Solution (HBSS) for 2.5 hours with agitation. Digests were pipetted 10 times every hour. Cells were pelleted at 1500 rpm for 5 min and resuspended in PBS. Cells were pelleted at 1500 rpm for 1 min and resuspened in PBS two more times the remaining cells were used for DNA extraction. PCR was carried out under standard conditions with three primers to detect the floxed, wildtype and deleted allele as described previously in [26].

### Whole mount staining

Whole mount staining was done as previously described [32]. The inguinal #4 mammary glands were removed and placed in Carnoy’s fixative for 1 hour and stained with Carmine Aluminum Stain overnight then dehydrated through graded ethanol, cleared with xylene, and mounted between two microscope slides.

### Histology

Mammary glands were removed and fixed in 4% paraformaldehyde for 1 hour at room temperature. Next, they were washed in PBS and dehydrated through graded ethanol, cleared with xylene and embedded in paraffin. Mammary glands were embedded in paraffin and 5-7 μm thick sections were cut. Hematoxylin and eosin staining was executed using the manufacturers instructions (Sigma-Aldrich).

### Mammosphere cultures

Mammary epithelial cells for mammosphere culture were isolated similarly to others [33]. Briefly, digested cells were pelleted at 1500 rpm for 5 min and resuspened in 8 ml .8% NH_4_Cl 10 mM EDTA and 2 ml HBSS supplemented with 2%FBS with a final concentration of 15 mM Hepes (HF) and centrifuged again for 5 min. Cells were then resuspended for 90 seconds with constant pipetting in 0.25% Trypsin EDTA and then pelleted by centrifugation. Pellets were then resuspended for 90 seconds with constant pipetting in 5 mg/ml Dispase (Gibco) and centrifuged. Finally, cells were diluted in 10 ml cold HF and filtered through 40 μm mesh. The cells were then plated in 6 well ultra low attachment cell culture plates at a concentration of 1,000-50,000 cells/well.

### Adenovirus infection

Single cells isolated for mammosphere cultures were plated at 25,000 cells/ml in monolayer or in suspension and infected 24 hours after plating with either Adenovirus containing Cre Recombinase with an IRES GFP and or a control Adenovirus GFP at an MOI of 200. 90-95% of cells were infected after 48 hours as assessed by presence of GFP.

### Whole gland transplants

Scid mice were used as transplant recipients. A small incision was made on fat pad in the interscapular region, a 25G needle was used to score the fat to stimulate blood flow and encourage grafting [34,35]. The #4 mammary glands were removed from Prx1-Cre; *Ift88*^Del^ and controls and placed on the scored area and stitched at the top and bottom. Glands were removed 4-6 weeks after transplantation and processed for whole mount carmine aluminum staining.

## Results

### Spatial activity of Cre and disruption of PC

**Figure 1:**
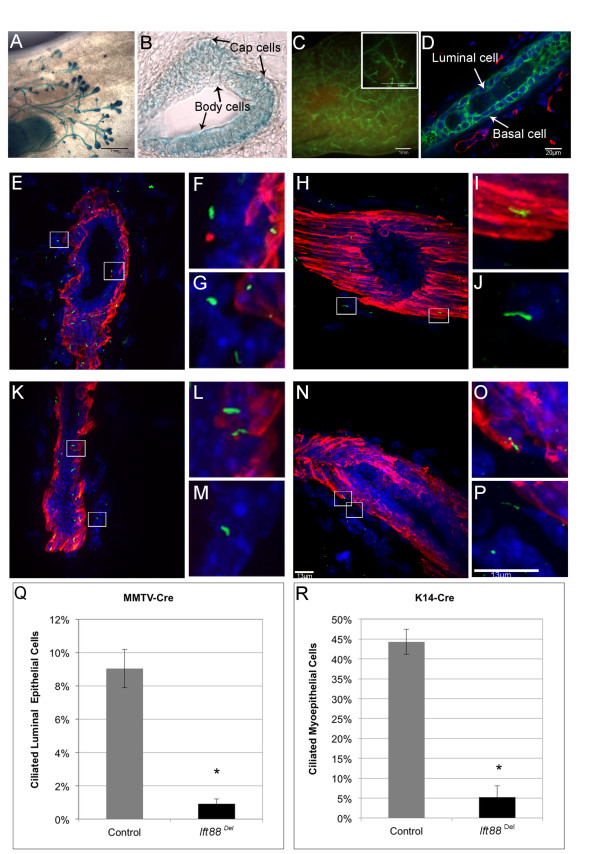
**Disruption of Primary Cilia in Mammary Epithelium. (A-D)** Cre reporter activity*.* X-gal stained mammary glands from 6-week-old MMTV-Cre; ROSA26^LacZ^ mice **(A, B)**. Whole mount x–gal staining **(A)**. Cryosection through an end bud of the gland shown in A **(B)**. Fluorescence in K14-Cre;ROSA26^mTmG^ adult mice **(C, D)**. Whole mount image **(C)** and a cryosection **(D)** are shown. **(E-P**) Immunofluorescent detection of PC in mammary gland. Thick cryosections of adult mammary tissue from MMTV-Cre control **(E-G)**, MMTV-Cre; *Ift88*^Del^ (K-M), K14-Cre control **(H-J)** and K14-Cre; *Ift88*^Del^**(N-P)** were stained with an antibody to the cilia specific protein, Arl13b (green). Myoepithelial cells were marked using an antibody to alpha Smooth Muscle Actin (red). Nuclei were stained blue. Areas shown in high magnification are boxed in the low magnification image to the left. Representative images of PC on luminal and myoepithelial **(F)**; stromal and myoepithelial **(G)**; myoepithelial **(I)**; stromal **(J)**; myoepithelial **(L)**; stromal **(M)**; myoepithelial **(O)**; and stromal cells **(P)** are shown, scale bars are 13 μm. Quantification of PC on luminal epithelial cells in MMTV-Cre; *Ift88*^Del^ mice **(Q)** and myoepithelial cells on K14-Cre; *Ift88*^Del^ mice **(R)** are shown.

We initially proposed that depletion of PC in mammary epithelium would yield phenotypes similar to those in mice with either activating or inactivating mutations in Hedgehog signaling. To test this hypothesis, we generated MMTV-Cre; *Ift88*^Del^ and K14-Cre; *Ift88*^Del^ mice. Before we characterized the mice, we confirmed that the Cre-models were working and that PC were depleted in the mammary gland. First, the spatial activity of Cre recombinase in the MMTV-Cre and K14-Cre lines was determined by crossing to Cre reporter strains, Gt(ROSA)26Sor^tm1Sor^/J (Rosa26^LacZ^) and Gt(ROSA)26Sor^tm4(ACTB-tdTomato,-EGFP)Luo^/J (ROSA26^mTmG^) respectively (Figure 1A-D). MMTV-Cre showed activity, as demonstrated by blue beta-galactosidase staining, throughout the mammary ducts. Luminal cells as well as body cells of the TEBs were more efficiently targeted than basal cells in mice at 5-weeks of age (Figure 1A, B) as described previously [27]. K14-Cre demonstrated activity (green membrane fluorescence) throughout the ducts of adult mice with both basal and luminal cells targeted (Figure 1C, D). We could not detect cells within ducts, basal or luminal, with red membrane staining indicating virtually every epithelial cell was targeted by K14-Cre (Figure 1D). Stromal cells were stained red and thus were not targeted by K14-Cre as expected (Figure 1D).

Deletion of the *Ift88* gene in MMTV-Cre and K14-Cre mice was confirmed by PCR of genomic DNA isolated from mammary epithelial cells from adult K14-Cre; *Ift88*^Del^ and MMTV-Cre; *Ift88*^Del^ mice as well negative and positive controls (data not shown). Disruption of PC in *Ift88*^Del^ mammary glands was determined by immunofluorescent staining of PC in cryosections from adult mammary glands using the cilia specific antibody Arl13B (Figure 1E-P; green). Myoepithelial cells were stained using an antibody to alpha smooth muscle actin (αSMA, red). The percentage of ciliated luminal epithelial cells (negative for αSMA) and the percentage of ciliated myoepithelial cells (positive for αSMA) were determined in control and MMTV-Cre and K14-Cre; *Ift88*^Del^ mammary glands, respectively (Figure 1Q, R). In agreement with previous results [36], PC were enriched on myoepithelial cells relative to luminal epithelial cells in control mice. Forty-four percent of myoepithelial cells observed contained a PC whereas only 9% of luminal cells had a PC. PC were significantly depleted in both MMTV-Cre and K14-Cre; *Ift88*^Del^ glands. The percentage of luminal cells that contained PC dropped from 9% in controls to 0.9% in MMTV-Cre; *Ift88*^Del^ mice (p < 0.01). The percentage of myoepithelial cells that contained PC dropped from 44% in controls to 5% in K14-Cre; *Ift88*^Del^ mice (p < 0.01). The results indicate that our Cre models are functional and that PC are significantly depleted in mammary epithelial cells.

### Characterization of mammary development in PC depleted mice

**Figure 2:**
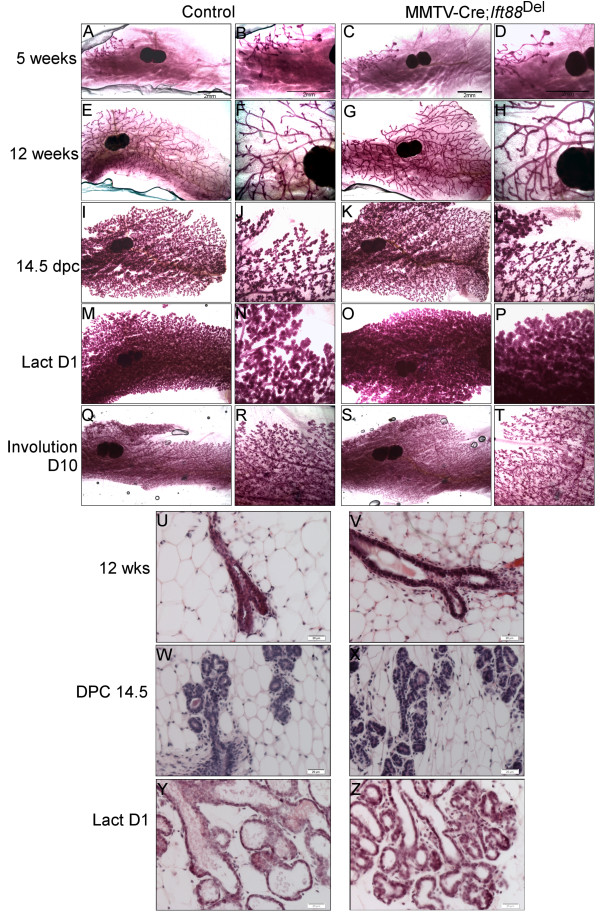
**Loss of *Ift88 *** **in MMTV-Cre expressing cells does not affect mammary development.** Whole mount carmine staining of #4 mammary glands. MMTV-Cre; *Ift88*^Del^ mice C,D,G,H,K,L,O,P,S,T) and controls (A,B,E,F,I,J,M,N,Q,R) at 5 wks **(A-D)**, 12 wks **(E-H)**, Pregnancy day 14.5 **(I-L)**, Lactation Day 1 **(M-P)**, and Involution day 10 **(Q-T)**. H&E staining of paraffin embedded sections from control **(U, W, Y)** and MMTV-Cre; *Ift88*^Del^**(V, X,Z)** mice at 12 weeks **(U,V)**; 14.5 days pregnancy **(W,X)**, and Day 1 lactation **(Y,Z)** are shown.

**Figure 3:**
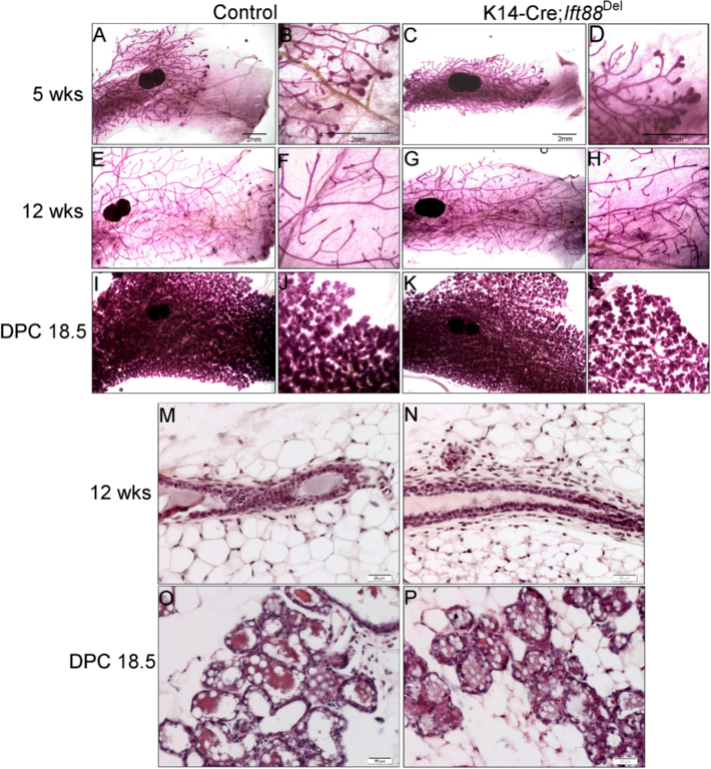
**Loss of *Ift88 *** **in K14-Cre expressing cells does not affect mammary development.** Whole mount carmine staining of #4 mammary glands from K14-Cre; *Ift88*^Del^ mice (C,D,G,H,K,L) and controls (A,B,E,F,I,J) at 5 weeks **(A-D)** and 12 weeks **(E-H)** of age and 18.5 days pregnant **(I-L)**. H&E staining of paraffin embedded sections from control **(M,O)** and K14-Cre; *Ift88*^Del^**(N, P)** mammary glands at 12 weeks (M,N) and day 18.5 pregnancy **(O,P)**.

Whole mount Carmine staining of glands and H&E stained sections from control and *Ift88*^Del^ mammary glands at varying stages of development were compared (Figures 2 and 3). MMTV-Cre; *Ift88*^Del^ glands did not show any detectable difference in branching or histological structure when compared to control mice through puberty and adulthood, 5 weeks to 6 months of age (Figure 2; n = 6 controls and 7 mutants). Pregnancy proceeded normally and the mice were able to lactate and support multiple litters. We did not detect alterations in mammary morphology or histology during pregnancy (n = 4 control and 4 mutant) or lactation (Figure 2; n = 4 control and 4 mutant). In addition, we did not detect tumors in whole mount preparations from mice aged between 8 and 18 months, including retired breeders (n = 9 controls and 10 mutants; data not shown).

Recently, it was shown that Hedgehog responding cells in the mammary gland are basally located, contain primary cilia, and express K14 [37]. Nevertheless, K14-Cre; *Ift88*^Del^ glands were not distinguishable from control glands through puberty and adulthood (Figure 3; n = 5 controls and 4 mutants) or during pregnancy and lactation (Figure 3; n = 3 controls and n = 2 mutants). These mice were also able to lactate and fully support multiple litters. Palpable tumors were not detected in any mice over 9 months of age. These mice did not demonstrate any histological phenotypes typical of mammary glands from mice with mutations in Hedgehog associated genes including *Gli2*^*-/-*^, *Ptch1Δ*^*-/+*^, *Ptch1*^mes^/*Ptch1*^mes^, MMTVrtA-*Gli1*, MMTV, or SmoM2 MMTV Cre knockin; which resulted in at least one or more defects including ductal dysplasia, distended ducts, hyperplasia, altered differentiation, excessive budding, or aberrant termini [16,38-40,47] (Figure 3 M-P and data not shown). The results indicate PC in MMTV- and K14- expressing lineages are not required for normal mammary development or function and loss of PC does not phenocopy mice with alterations in Hedgehog signaling proteins.

### PC are enriched in mammospheres

**Figure 4:**
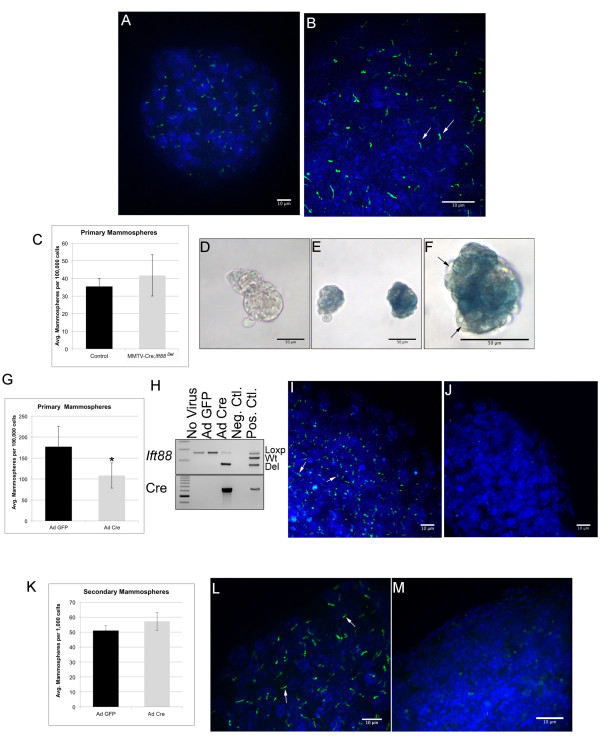
**Mammospheres are enriched for Primary Cilia.** Mammosphere cultures derived from wild type mammary glands were immunofluorescently stained for PC **(A, B)** using anti-Arl13B antibody (green). Nuclei were stained with DAPI (blue). Low **(A)** and high **(B)** magnification images are shown. The number of mammospheres generated from equal numbers of mammary epithelial cells isolated from control and MMTV-Cre; *Ift88*^Del^ glands were determined **(C)**. Mammospheres derived from control, Cre-negative **(D)** and MMTV-Cre; ROSA26^LacZ^**(E,F)** glands were stained with ß-galactosidase (blue). Mammospheres demonstrated variable levels of staining **(E)** and staining within mammospheres was chimeric (**F**; arrows, representative unstained cells). Mammary epithelial cells from *Ift88*^LoxP/LoxP^ mice were infected with Ad-GFP or Ad-Cre adenovirus and the number of mammospheres that grew from equal number of cultured cells was determined **(G)**. Deletion of *Ift88* was determined in genomic DNA isolated from the cells in mammosphere using PCR to detect Cre and the LoxP, wild type (Wt), and deleted (Del) alleles **(H)**. PC were stained using Arl13B antibody (green) in Ad-GFP **(I)** and Ad-Cre **(J)** infected mammospheres. Nuclei were stained with DAPI (Blue). Ad-GFP and Ad-Cre infected mammospheres were dissociated and plated into secondary mammosphere culture and the number of mammospheres generated was counted **(K)**. PC were stained in Ad-GFP **(L)** and Ad-Cre **(M)** infected secondary mammopheres using Arl13B antibody (green) and DAPI (blue).

It was recently shown that ectopic Hedgehog signaling in the mammary gland induces the expansion of basal cells that contain PC [37]. It is not clear if MMTV-Cre or K14-Cre would have targeted this small population of stem or progenitor cells in vivo so to determine if PC regulate outgrowth of mammary stem and progenitor cells we used a mammosphere culture model. Mammospheres are an aggregate of non-adherent mammary epithelial cells clonally derived from stem and progenitor cells [33]. Mammospheres are a way to enrich this population of cells for experimentation in culture. We hypothesized that loss of PC would affect outgrowth or renewal of mammospheres via regulation of mammary stem and progenitor cells. First, we grew mammary epithelial cells from wild type mice in mammosphere culture and stained for PC using the Arl13B antibody. In contrast to what we observed in whole mammary gland, where PC are found on a limited number of cells, virtually every cell in the mammosphere demonstrated a PC (Figure 4A, B) suggesting PC are enriched on mammary stem and progenitor cells although culture conditions could contribute to the formation of PC. Next, we grew mammary cells from MMTV-Cre; *Ift88*^Del^ and Cre-negative controls in mammosphere culture and measured the number of mammospheres that grew out from 4 × 10^4^ cells over 10 days. Only spheres over 50 microns were scored. No statistical difference was detected in the number of mammospheres in MMTV-Cre; *Ift88*^Del^ versus control cultures (Figure 4C n = 3). To determine if MMTV-Cre was active in the stem or progenitor cells that make-up the mammospheres, we grew mammary epithelial cells isolated from Cre-negative and MMTV-Cre; Rosa 26^LacZ^ females in mammosphere culture and stained for beta-galactosidase activity (blue, Figure 4D-F). Mammosphere staining was mosaic suggesting that MMTV-Cre may have become active after the mammosphere formed. In this case the initiating stem or progenitor cell may not have been targeted by MMTV-Cre in vivo.

Next, to achieve more widespread depletion of PC in mammosphere cultures, we used an adenovirus expressing Cre with IRES controlled GFP (Ad-CRE) or a control GFP only virus (Ad-GFP) to infect mammary epithelial cells isolated from Ift88^LoxP/LoxP^ mice. Infected cells were then placed in mammosphere culture at 5 × 10^4^ cells per well for 10 days (Figure 4G). Deletion of *Ift88* was confirmed by PCR of genomic DNA isolated from the Ad-Cre and Ad-GFP infected cells (Figure 4H). Widespread depletion of PC was confirmed using immunofluorescent staining (Figure 4I,J). There was a significant 50% decrease in the formation of primary mammospheres in the PC-depleted cultures (Figure 4G; n = 4 separate experiments p-value < 0.05 paired *T*-test). However, when the mammospheres were passaged into secondary mammosphere cultures (1 × 10^4^ cells/well) to test for self-renewal we did not detect statistically significant differences in outgrowth of mammospheres (Figure 4K n = 2 separate experiments) although PC were still clearly depleted from the secondary cultures when compared to the controls (Figure 4L,M). PC may affect the initial outgrowth of mammospheres, but they are not required for renewal of the cultures.

### The mammary phenotype in Prx-1-Cre; Ift88^Del^ mice is not inherent to the mammary gland

**Figure 5:**
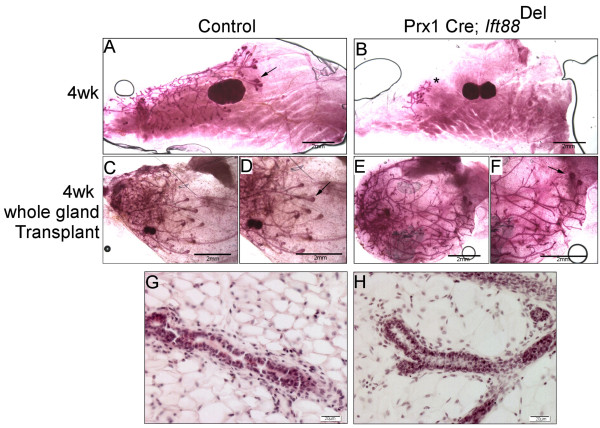
**The mammary phenotype in Prx1-Cre; *Ift88***^**Del **^**mice is likely due to systemic factors.** Whole mount Carmine staining of #4 mammary glands from 4 week old control **(A)** and Prx1-Cre; *Ift88*^Del^**(B)** mice. Control **(C,D)** and Prx1- Cre; *Ift88*^Del^**(E,F)** mammary glands were transplanted onto 3 week old *Ift88*- positive Scid mice. Transplants were removed after 4 weeks and stained with Carmine. Low **(C,E)** and high **(D,F)** magnification images are shown. Representative terminal end buds are marked with arrows **(D,F)**. Carmine stained whole mounts from control **(G)** and Prx1-Cre; *Ift88*^Del^**(H)** transplants were then sectioned and stained with H&E.

Mammary development is a highly controlled event with all cell types working together. Coordinated crosstalk between epithelial cells and stroma allows for proper terminal end bud formation, ductal extension, and side branching [2,41]. Mice with LacZ knocked in to the *Ift88* locus were found to have high *Ift88* expression in the mammary stroma and studies with ROSA26^LacZ^ reporter mice showed that Prx1-Cre is active in the mammary stroma as well as ovaries and other tissues [24]. We showed previously and confirm here that Prx1-Cre; *Ift88*^Del^ mice have defective TEB formation and delayed ductal elongation [24]; Figure 5A,B). We proposed that the mammary phenotype was indirect and mediated by alterations in the ovary since exogenous estrogen injection could restore end bud activity [24]. To test whether the loss of PC in the mammary stroma affects mammary development without the confounding effects of disrupted ovarian hormone levels, we transplanted whole mammary glands from 3 week-old Prx1-Cre; *Ift88*^Del^ and littermate controls mice onto the highly vascularized region between the shoulder blades of Scid hosts similar to what was described in [34]. Transplants were removed after 4 weeks and stained with carmine; sections were stained with H&E (Figure 5C, D); n = 3 controls and 4 mutants). Transplants exhibited typical terminal end bud formation (Figure 5C-F) as well as normal ductal histology (Figure 5G, H), indicating that the phenotype observed in Prx1-Cre; *Ift88*^Del^ mice is not inherent to the mammary gland.

### TEBs and mammary branching are normal in Ift88^Orpk/Orpk^ female mice on Bl/6 or Balbc background

**Figure 6:**
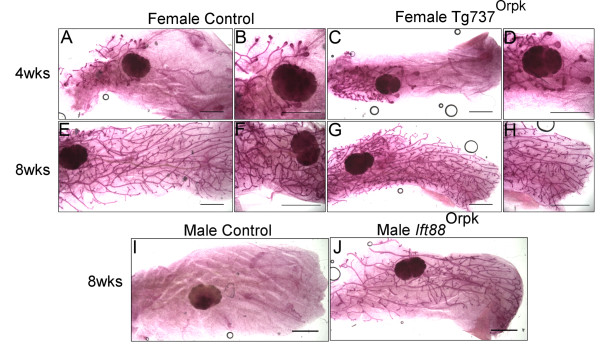
**Mammary phenotype in *Ift88***^**Orpk **^**mice on Bl/6 and Balb/c background.** Mammary glands from female mice **(A-H)**. Whole mount carmine staining of #4 mammary glands in 4 week **(A-D)** and 8 week **(E-H)** old females from control **(A, B, E, F)** and *Ift88*^Orpk^ (C, D, G, H; Bl/6 shown) mice. Mammary glands from male mice **(I, J)**. Whole mount Carmine staining of #4 mammary glands in 8 week old males from control **(I)** and *Ift88*^Orpk^ (L; Bl/6 shown).

In an effort to study mice that had reduced ciliary function in epithelial and stromal compartments of the mammary gland, we examined mammary glands in the well-characterized Oak Ridge Polycystic kidney disease (*Ift88*^Orpk^) mouse model. Orpk is a hypomorphic allele of *Ift88* and thus *Ift88* has some function in these mice. *Ift88*^Orpk^ females from both Bl/6 and Balb/c backgrounds showed no obvious developmental deviations from controls as determined by whole mount staining (Figure 6A-H; n = 6 control, 4 mutant). This is in contrast to a previous report of *Ift88*^Orpk^ mice on a mixed C57Bl/6 × C3H/FVB background, in which a decrease in ductal extension and branching was noted [36]. Together the results suggest that there may be a strong genetic modifier effect on PC function in the mammary gland.

Male Bl/6 and Balb/c mice do not generally develop a mammary ductal tree. During embryonic development, mesenchymal cells, in response to testosterone, condense around the stalk of the developing mammary bud, severing the connection of the bud to the surface epidermis. The epithelial cells subsequently undergo apoptosis and thus a ductal tree is not formed [42]; and Figure 6I). In contrast, male *Ift88*^Orpk^ developed a complete ductal tree by 8 weeks of age in both Bl/6 and Balb/c backgrounds (Figure 6I,J; n = 3 controls and 5 mutants). The results suggest that *Ift88* is somehow required to prevent mammary ductal development in male mice.

## Discussion

We developed mouse models with deletion of PC in mammary epithelial or myoepithelial cells for evaluation of the function of PC in mammary development. We confirmed that *Ift88* was deleted in Cre-positive mice and that PC were disrupted in the expected cell types. Nevertheless, minimal disruption to normal mammary development was observed and mice were able to feed and support several litters of pups. In addition, tumors were not detected in mice aged out to 18 months. Mammary glands from virgin mice with a hypomorphic allele of *Ift88*, *Ift88*^Orpk^, did not display defects in branching morphogenesis in adult mice; however, males from this line of mice demonstrated uncharacteristic development of a full ductal tree. While characterizing the PC deleted mice, we discovered that PC were enriched on cells grown in mammospheres. Deletion of *Ift88* in these cells using an Adenovirus Cre resulted in reduced expansion of cells into primary mammospheres; however, outgrowth of secondary mammospheres was not affected. We also used whole gland transplants to show that delayed mammary development observed in Prx1-Cre; *Ift88*^Del^ mice, in which the mammary stroma and other tissues are targeted, is likely due to systemic factors and not inherent to the mammary gland.

Our original hypothesis when initiating these studies was that deletion of *Ift88* and subsequent depletion of PC would phenocopy alterations in mammary development observed in mice with disruptions to Hh signaling. It is well known that PC mediate signaling by Hh proteins in many cell types [9]. In addition to regulating ligand dependent signaling, PC are required to process Gli3 to a repressor form. Active Hh signaling in the epithelium does not appear to be required for normal mammary development although there is evidence to suggest Hh signaling in the stroma may be required. Deletion of Gli2 in mice results in ductal dysplasia and extended ducts; however, when Gli2 null epithelium was transplanted in to a normal host fat pad development was normal suggesting a role for Hh signaling in the stroma [16]. We previously showed that Prx1-Cre has some activity in the mammary stroma. Unfortunately, Prx1 is also active in additional tissues throughout the mouse including the ovary [24]. To alleviate the confounding effects of PC loss in the ovary, we transplanted mammary glands from Prx1-Cre; *Ift88*^Del^ mice to wild type hosts. The formation of TEBs and branching appeared normal in the transplanted tissue suggesting the effects seen in the Prx1-Cre; *Ift88*^Del^ mice were due to systemic factors.

While loss of Hh signaling in mammary epithelium does not appear to affect normal development, inappropriate activation of Hh signaling via an activated smoothened protein in mammary epithelium results in excessive budding in TEBs, increased proliferation of epithelial cells, and alterations in luminal cell differentiation suggesting that repression of Hh signaling plays an important role in normal mammary development [18,43,44]. Based on these data we predicted that loss of PC, which would be expected to inhibit the formation of the Gli3 repressor, would mimic phenotypes seen in mice with inappropriate activation of Hh signals in epithelial cells; however, we did not detect any significant alterations in development of MMTV-Cre or K14-Cre; *Ift88*^Del^ mammary glands. One explanation for normal development in absence of PC is that there may be a constant level of Hh signal activation in the mammary gland that is continuously repressed by Gli3 to allow normal development and prevent tumor formation. In the absence of cilia, this positive signal would not exist so Gli3 repressor not required. Loss of Gli3 results in failure in the formation of specific mammary buds, but the role of the Gli3 repressor in post-natal mammary development was not addressed [15].

Research involving PC and their relationship with cancer has become an area of intense interest. Recently, the involvement of PC in basal cell carcinoma and medulloblastoma were investigated [19,20]. It was shown that PC can work to prevent tumorigenesis in some contexts but promote it in others. If the hedgehog pathway was activated at the level of Smoothened, the presence of PC accelerated tumor formation; however, with the loss of PC, tumor formation was suppressed. Suppression was likely due to the fact that PC are required for Smoothened function. On the other hand, if tumors were initiated with a constitutively active form of Gli2, which would be downstream of Smoothened function, loss of cilia accelerated tumor growth [19,20]. Accelerated growth was due to loss of the Gli3 repressor, which would counteract the functions of Gli2. For breast cancer, it has been shown that the incidence of PC decreases during the progression of normal to cancerous cells, independent of the levels of proliferation, leading to the hypothesis that PC act as tumor suppressors [21,45]. Even though we did not detect significant alterations in mammary development or spontaneous tumors in aged mice, our results do not rule out an effect of PC on breast cancer in combination with other signals. For example, loss of PC in breast cancers initiated by Gli1 may promote tumor formation whereas tumors initiated by excess Hh ligand, mutations in Ptc1, or activated Smoothened could be inhibited.

A role for Hh signaling in mammary stem cells has been suggested. Mammary stem cells cultured as mammospheres demonstrate high levels of markers for Hh signaling including Ptch1 and Gli1 [46]. Furthermore, activated Smoothened resulted in increased mammosphere formation suggesting Hh may positively regulate stem cell populations in the mammary gland [47]. Misexpression of Shh in the mammary glands of transgenic mice results in ductal dysplasia after pregnancy [37]. Hh responding cells were localized by Ptch1-LacZ staining to the basal layer within the dysplastic areas. Hh responding cells were ciliated and expressed markers of basal and stem/progenitor cells suggesting PC may have a positive role in regulation of mammary stem and progenitor populations. In agreement, we show enrichment of ciliated cells in mammosphere cultures. Although It is possible culture conditions necessary for mammosphere formation may promote PC formation, this observation confirms the previous results described above in which cells in vivo expressing progenitor markers are enriched for PC [37]. We also show a decrease in outgrowth of primary mammospheres that could be due to loss of the positive Hh signal due to absence of PC in the mammosphere initiating cells. However, we do not see an effect of losing PC on passage of the cultures into secondary mammospheres suggesting PC do not play a role in renewal of cells in the established mammospheres.

Recently, it was shown that mice with a germline, hypomorphic allele of *Ift88*, *Ift88*^Orpk^, have defects in mammary development [36]. Branching morphogenesis during puberty and alveolar differentiation during pregnancy were inhibited. Epithelium from the *Ift88*^Orpk^ transplanted into cleared fat pads also demonstrated inhibition of branching suggesting the phenotype was due to loss of PC in the mammary epithelium and not due to systemic effects. Decreased Hh signaling and increased canonical Wnt signaling were noted although neither a decrease in Hh signaling nor an increase in canonical Wnt signaling would be expected to result in reduced branching. We did not observe alterations in branching morphogenesis in *Ift88*^Orpk^ mice in our colony. One explanation for the different observations is the background strains used for each study. The *Ift88*^Orpk^ mice in our colony are on the C57Bl/6 or Balb/C backgrounds. The mice used in the previous study were F1 hybrids generated by crossing *Ift88*^Orpk^ mice on the C57Bl/6 background to *Ift88*^Orpk^ mice on a mixed C3H and FVB background. Together the results suggest a strong genetic modifier that affects the function of PC and their role in the mammary phenotype.

Mammary buds are formed in the embryos of both male and female mice; however, in most mouse strains, male mice do not develop a ductal tree [48]. Androgen receptors are found in the mammary mesenchyme starting at embryonic day 13. In response to testosterone production in the male embryos, this mesenchyme condenses around the epithelial bud, separating it from the external ectoderm and triggering regression of the epithelium. The time window for this process is between embryonic day 13 and 16. While characterizing mammary glands in *Ift88*^Orpk^ mice, we discovered that male mice carrying this hypomorphic allele of *Ift88* have a fully developed mammary ductal tree. The mechanism of this alteration is not known and could involve local PC in the mammary mesenchyme or PC in other tissues that would have systemic effects on regression of the nascent mammary duct. Recently, it was shown that male embryos with a mutation in Gli3 (Gli3^xt/xt^) demonstrate inappropriate retention of mammary buds. Furthermore, misactivation of Hh signaling through expression of Gli1 resulted in a similar phenotype. It was suggested that repression of Hh signal through Gli3 repressor activity is required for proper specification of the mammary mesenchyme. Abnormal mammary mesenchyme including low levels of the androgen receptor likely prevented the destruction of the mammary bud in mutant male embryos [49]. Since PC are required for processing of the Gli3 repressor, it is possible that altered PC function in the mammary mesenchyme acts through a similar mechanisms.

PC also regulate pathways other than Hh including Wnt, PDGF, and von-Hippel Lindau tumor suppressor [4,7,9,10]. Wnt proteins are well established as key regulators of mammary development, misexpression of canonical Wnt/ß-catenin signaling in the mammary epithelium results in increased branching, lobuloalveolar hyperplasia and tumorigenesis; whereas inactivation of canonical signaling results in a reduction in branching and TEB number [50,51]. Loss of a noncanonical signaling Wnt, Wnt5a, resulted in rapid ductal elongation and increased side branching, while misexpression of Wnt5a resulted in a lactation defect [31,52]. Even though Wnt signaling is key to mammary development, recent data suggest PC do not directly regulate Wnt/ß-catenin signaling [53,54]. Most importantly, we did not detect any phenotypes characteristic of alterations in Wnt/ß-catenin signaling in our PC-deleted mouse models. PDGFR-alpha is localized to the PC; however studies describing the loss of this receptor in the mammary gland have not been reported [55]. We also did not detect any indication of alterations in signaling by the tumor suppressor von- Hippel Lindau. Its loss in the mammary epithelium causes reduced proliferation and alveolar differentiation during pregnancy but does not cause tumorigenesis [56].

## Conclusion

We conclude that PC can regulate mammary development through systemic factors, but, overall, PC localized in the gland have a limited role in development or normal function of the mammary gland.

## Abbreviations

PC: Primary cilia; TEB: Terminal end bud; IFT: Intraflagellar transport; Hh: Hedgehog; ROSA26: Reverse oriented splice acceptor β-galactosidase/neomycin 26; Ad: Adenovirus; PFA: Paraformaldehyde; DAPI: 4′,6-diamidino-2-phenylindole; PBS: Phosphate buffered saline; PCR: Polymerase chain reaction; Del: Deleted; WT: Wild type; GFP: Green fluorescent protein; IRES: Internal Ribosome entry site; Orpk: Oak Ridge Polycystic Kidney disease; HBSS: Hanks balanced salt solution; HEPES: 4-(2-Hydroxyethyl)piperazine-1-ethanesulfonic acid sodium salt; FBS: Fetal bovine serum; HF: HBSS plus 15 mM HEPES and 2% FBS; X-GAL: 4-chloro-5-bromo-3-indoyl-β-D-galactopyranoside; EDTA: Ethylenediaminetetraacetic acid.

## Competing interests

The authors declare that they have no competing interests.

## Authors’ contributions

RS and EHM designed experiments. EHM performed experiments. EHM and RS wrote the manuscript. All the authors read and approved of the final version of the manuscript.

## References

[B1] Wiseman BS Werb Z Stromal effects on mammary gland development and breast cancer Science 2002 296 1046 1049 10.1126/science.1067431 12004111 PMC2788989

[B2] Hynes NE Watson CJ Mammary gland growth factors: roles in normal development and in cancer Cold Spring Harb Perspect Biol 2010 2 a003186 20554705 10.1101/cshperspect.a003186 PMC2908768

[B3] Pedersen LB Veland IR Schrøder JM Christensen ST Assembly of primary cilia Dev Dyn 2008 237 1993 2006 10.1002/dvdy.21521 18393310

[B4] Berbari NF O’Connor AK Haycraft CJ Yoder BK The primary cilium as a complex signaling center Curr Biol 2009 19 R526 R535 10.1016/j.cub.2009.05.025 19602418 PMC2814769

[B5] D’Angelo A Franco B The dynamic cilium in human diseases PathoGenetics 2009 2 3 10.1186/1755-8417-2-3 19439065 PMC2694804

[B6] Singla V The Primary Cilium as the Cell’s Antenna: Signaling at a Sensory Organelle Science 2006 313 629 633 10.1126/science.1124534 16888132

[B7] Veland IR Awan A Pedersen LB Yoder BK Christensen ST Primary Cilia and Signaling Pathways in Mammalian Development, Health and Disease Nephron Physiol 2009 111 39 53 10.1159/000208212 PMC2881330 19276629

[B8] Badano J Mitsuma N Beales P Katsanis N The ciliopathies: an emerging class of human genetic disorders Annu Rev Genomics Hum Genet 2006 7 125 10.1146/annurev.genom.7.080505.115610 16722803

[B9] Nielsen SK Møllgård K Clement CA Veland IR Awan A Yoder BK Novak I Christensen ST Characterization of primary cilia and hedgehog signaling during development of the human pancreas and in human pancreatic duct cancer cell lines Dev Dynam 2008 237 2039 2052 10.1002/dvdy.21610 18629868

[B10] Christensen ST Pedersen SF Satir P Veland IR The primary cilium coordinates signaling pathways in cell cycle control and migration during development and tissue repair Current topics in Developmental Biology 2008 85 261 301 19147009 10.1016/S0070-2153(08)00810-7

[B11] Hatsell S Frost AR Hedgehog signaling in mammary gland development and breast cancer Annu Rev Genomics Hum Genet 2007 12 163 173 10.1007/s10911-007-9048-2 17623270

[B12] Liu A Wang B Niswander LA Mouse intraflagellar transport proteins regulate both the activator and repressor functions of Gli transcription factors Development 2005 132 3103 3111 10.1242/dev.01894 15930098

[B13] Wang Y Zhou Z Walsh CT McMahon AP Selective translocation of intracellular Smoothened to the primary cilium in response to Hedgehog pathway modulation Proc Natl Acad Sci 2009 106 2623 2628 10.1073/pnas.0812110106 19196978 PMC2650314

[B14] Tukachinsky H Lopez LV Salic A A mechanism for vertebrate Hedgehog signaling: recruitment to cilia and dissociation of SuFu-Gli protein complexes J Cell Biol 2010 191 415 428 10.1083/jcb.201004108 20956384 PMC2958481

[B15] Hatsell SJ Cowin P Gli3-mediated repression of Hedgehog targets is required for normal mammary development Development 2006 133 3661 3670 10.1242/dev.02542 16914490

[B16] Lewis MT Ross S Strickland PA Sugnet CW Jimenez E Hui C Daniel CW The Gli2 transcription factor is required for normal mouse mammary gland development Dev Biol 2001 238 133 144 10.1006/dbio.2001.0410 11783999

[B17] May Yin Lee LSJMV Hedgehog and Gli Signaling in Embryonic Mammary Gland Development J Mammary Gland Biol Neoplasia 2013 18 133 10.1007/s10911-013-9291-7 23677624 PMC3691482

[B18] P Visbal A T Lewis M Hedgehog Signaling in the Normal and Neoplastic Mammary Gland Curr Drug Targets 2010 11 1103 1111 10.2174/138945010792006753 20545610 PMC5499530

[B19] Wong SY Seol AD So P-L Ermilov AN Bichakjian CK Epstein EH Dlugosz AA Reiter JF Primary cilia can both mediate and suppress Hedgehog pathway–dependent tumorigenesis Nat Med 2009 15 1055 1061 10.1038/nm.2011 19701205 PMC2895420

[B20] Han Y-G Kim HJ Dlugosz AA Ellison DW Gilbertson RJ Alvarez-Buylla A Dual and opposing roles of primary cilia in medulloblastoma development Nat Med 2009 15 1062 U114 10.1038/nm.2020 19701203 PMC2771737

[B21] Yuan K Frolova N Xie Y Wang D Cook L Kwon Y-J Steg AD Serra R Frost AR Primary cilia are decreased in breast cancer: analysis of a collection of human breast cancer cell lines and tissues J Histochem Cytochem 2010 58 857 870 10.1369/jhc.2010.955856 20530462 PMC2942739

[B22] Seeley ES Carriere C Goetze T Longnecker DS Korc M Pancreatic Cancer and Precursor Pancreatic Intraepithelial Neoplasia Lesions Are Devoid of Primary Cilia Cancer Res 2009 69 422 430 10.1158/0008-5472.CAN-08-1290 19147554 PMC2629528

[B23] Moser JJ Fritzler MJ Rattner JB Primary ciliogenesis defects are associated with human astrocytoma/glioblastoma cells BMC Cancer 2009 9 448 10.1186/1471-2407-9-448 20017937 PMC2806408

[B24] Johnson ET Nicola T Roarty K Yoder BK Haycraft CJ Serra R Role for primary cilia in the regulation of mouse ovarian function Dev Dyn 2008 237 2053 2060 10.1002/dvdy.21612 18629867

[B25] Choi YS Chakrabarti R Escamilla-Hernandez R Sinha S Elf5 conditional knockout mice reveal its role as a master regulator in mammary alveolar development: Failure of Stat5 activation and functional differentiation in the absence of Elf5 Dev Biol 2009 329 227 241 10.1016/j.ydbio.2009.02.032 19269284

[B26] Haycraft CJ Zhang Q Song B Jackson WS Detloff PJ Serra R Yoder BK Intraflagellar transport is essential for endochondral bone formation Development 2007 134 307 316 10.1242/dev.02732 17166921

[B27] Teissedre B Pinderhughes A Incassati A Hatsell SJ Hiremath M Cowin P MMTV-Wnt1 and -DeltaN89beta-catenin induce canonical signaling in distinct progenitors and differentially activate Hedgehog signaling within mammary tumors PLoS ONE 2009 4 e4537 10.1371/journal.pone.0004537 19225568 PMC2639708

[B28] Wagner K Ward T Davis B Wiseman R Spatial and temporal expression of the Cre gene under the control of the MMTV-LTR in different lines of transgenic mice Transgenic Research 2001 10 545 553 10.1023/A:1013063514007 11817542

[B29] O’Connor AK Malarkey EB Berbari NF Croyle MJ Haycraft CJ Bell P Hohenstein P Kesterson RA Yoder BK An inducible CiliaGFP mouse model for in vivo visualization and analysis of cilia in live tissue Cilia 2013 2 8 10.1186/2046-2530-2-8 23819925 PMC3700774

[B30] Caspary T Larkins CE Anderson KV The Graded Response to Sonic Hedgehog Depends on Cilia Architecture Dev Cell 2007 12 767 778 10.1016/j.devcel.2007.03.004 17488627

[B31] Roarty K Serra R Wnt5a is required for proper mammary gland development and TGF-{beta}-mediated inhibition of ductal growth Development 2007 134 3929 3939 10.1242/dev.008250 17898001

[B32] Rasmussen S Young LT Smith G Ip M, Asch B Preparing Mammary Gland Whole Mounts from Mice Methods in Mammary Gland Biology and Breast Cancer Research 2000 Springer US, Boston, MA 75–85–85. [Methods in Mammary Gland Biology and Breast Cancer Research]

[B33] Dontu G Abdallah WM Foley JM Jackson KW Clarke MF Kawamura MJ Wicha MS In vitro propagation and transcriptional profiling of human mammary stem/progenitor cells Genes Dev 2003 17 1253 1270 10.1101/gad.1061803 12756227 PMC196056

[B34] Ormerod EJ Rudland PS Regeneration of mammary glands in vivo from isolated mammary ducts J Embryol Exp Morphol 1986 96 229 243 3805986

[B35] DeOme KB Faulkin LJ BERN HA BLAIR PB Development of mammary tumors from hyperplastic alveolar nodules transplanted into gland-free mammary fat pads of female C3H mice Cancer Res 1959 19 515 520 13663040

[B36] McDermott KM Liu BY Tlsty TD Pazour GJ Primary Cilia Regulate Branching Morphogenesis during Mammary Gland Development Current Biology 2010 20 731 737 10.1016/j.cub.2010.02.048 20381354 PMC2916967

[B37] García-Zaragoza EE Pérez-Tavarez RR Ballester AA Lafarga VV Jiménez-Reinoso AA Ramírez AA Murillas RR Gallego MIM Intraepithelial paracrine Hedgehog signaling induces the expansion of ciliated cells that express diverse progenitor cell markers in the basal epithelium of the mouse mammary gland Dev Biol 2012 372 28 44 10.1016/j.ydbio.2012.09.005 23000969

[B38] Moraes RC Chang H Harrington N Landua JD Prigge JT Lane TF Wainwright BJ Hamel PA Lewis MT Ptch1 is required locally for mammary gland morphogenesis and systemically for ductal elongation Development 2009 136 1423 1432 10.1242/dev.023994 19297414 PMC2675781

[B39] Lewis MT Ross S Strickland PA Sugnet CW Jimenez E Scott MP Daniel CW Defects in mouse mammary gland development caused by conditional haploinsufficiency of Patched-1 Development 1999 126 5181 5193 10529434 10.1242/dev.126.22.5181

[B40] Fiaschi M Rozell B Bergström A Toftgard R Kleman MI Targeted expression of GLI1 in the mammary gland disrupts pregnancy-induced maturation and causes lactation failure J Biol Chem 2007 282 36090 36101 10.1074/jbc.M704280200 17928300

[B41] Polyak K Kalluri R The role of the microenvironment in mammary gland development and cancer Cold Spring Harb Perspect Biol 2010 2 a003244 20591988 10.1101/cshperspect.a003244 PMC2964182

[B42] Cowin P Wysolmerski J Molecular mechanisms guiding embryonic mammary gland development Cold Spring Harb Perspect Biol 2010 2 a003251 20484386 10.1101/cshperspect.a003251 PMC2869520

[B43] Lewis MT Hedgehog signaling in mouse mammary gland development and neoplasia J Mammary Gland Biol Neoplasia 2001 6 53 66 10.1023/A:1009516515338 11467452

[B44] Lewis MT Veltmaat JM Next stop, the twilight zone: hedgehog network regulation of mammary gland development J Mammary Gland Biol Neoplasia 2004 9 165 181 15300011 10.1023/B:JOMG.0000037160.24731.35

[B45] Yuan K Serra R Frost AR Primary Cilia in the Breast and Breast Cancer Open Breast Cancer J 2011 2 101 107

[B46] Liu S Dontu G Mantle ID Patel S Ahn N-S Jackson KW Suri P Wicha MS Hedgehog signaling and Bmi-1 regulate self-renewal of normal and malignant human mammary stem cells Cancer Res 2006 66 6063 6071 10.1158/0008-5472.CAN-06-0054 16778178 PMC4386278

[B47] Moraes RC Zhang X Harrington N Fung JY Wu M-F Hilsenbeck SG Allred DC Lewis MT Constitutive activation of smoothened (SMO) in mammary glands of transgenic mice leads to increased proliferation, altered differentiation and ductal dysplasia Development 2007 134 1231 1242 10.1242/dev.02797 17287253

[B48] Parmar H Cunha GR Epithelial-stromal interactions in the mouse and human mammary gland in vivo Endocr Relat Cancer 2004 11 437 458 10.1677/erc.1.00659 15369447

[B49] Chandramouli A Hatsell SJ Pinderhughes A Koetz L Cowin P Gli activity is critical at multiple stages of embryonic mammary and nipple development PLoS ONE 2013 8 e79845 10.1371/journal.pone.0079845 24260306 PMC3832531

[B50] Tsukamoto AS Grosschedl R Guzman RC Parslow T Varmus HE Expression of the int-1 gene in transgenic mice is associated with mammary gland hyperplasia and adenocarcinomas in male and female mice Cell 1988 55 619 625 10.1016/0092-8674(88)90220-6 3180222

[B51] Jardé T Dale T Wnt signalling in murine postnatal mammary gland development Acta Physiol (Oxf) 2011 204 118 127 21518264 10.1111/j.1748-1716.2011.02283.x

[B52] Baxley SE Jiang W Serra R Misexpression of Wingless-Related MMTV Integration Site 5A in Mouse Mammary Gland Inhibits the Milk Ejection Response and Regulates Connexin43 Phosphorylation Biology of Reproduction 2011 85 907 915 10.1095/biolreprod.111.091645 21753195 PMC3197912

[B53] Ocbina PJR Tuson M Anderson KV Primary Cilia Are Not Required for Normal Canonical Wnt Signaling in the Mouse Embryo PLoS ONE 2009 4 8 e6839 10.1371/journal.pone.0006839 19718259 PMC2729396

[B54] Tasouri E Tucker KL Primary cilia and organogenesis: is Hedgehog the only sculptor? Cell Tissue Res 2011 345 21 40 10.1007/s00441-011-1192-8 21638207

[B55] Schneider L Clement CA Teilmann SC Pazour GJ Hoffmann EK Satir P Christensen ST PDGFRalphaalpha signaling is regulated through the primary cilium in fibroblasts Curr Biol 2005 15 1861 1866 10.1016/j.cub.2005.09.012 16243034

[B56] Seagroves TN Peacock DL Liao D Schwab LP Krueger R Handorf CR Haase VH Johnson RS VHL deletion impairs mammary alveologenesis but is not sufficient for mammary tumorigenesis Am J Pathol 2010 176 2269 2282 10.2353/ajpath.2010.090310 20382704 PMC2861092

